# Mechanisms of Natural Extracts of *Andrographis paniculata* That Target Lipid-Dependent Cancer Pathways: A View from the Signaling Pathway

**DOI:** 10.3390/ijms23115972

**Published:** 2022-05-26

**Authors:** Ruth Naomi, Hasnah Bahari, Zhi Yi Ong, Yong Yoke Keong, Hashim Embong, Retnagowri Rajandram, Soo Huat Teoh, Fezah Othman, Rosnani Hasham, Khoo Boon Yin, Priyatharisni Kaniappan, Muhammad Dain Yazid, Zainul Amiruddin Zakaria

**Affiliations:** 1 Department of Human Anatomy, Faculty of Medicine and Health Sciences, Universiti Putra Malaysia, Serdang 43400, Malaysia; ruthmanuel2104@gmail.com (R.N.); haba@upm.edu.my (H.B.); yoke_keong@upm.edu.my (Y.Y.K.); 2 School of Psychology, University of New South Wales, Sydney 2052, Australia; zhiyi.ong@unsw.edu.au; 3 Department of Emergency Medicine, Faculty of Medicine, Universiti Kebangsaan Malaysia, Kuala Lumpur 56000, Malaysia; hashimembong77@ukm.edu.my; 4 Department of Surgery, Faculty of Medicine, Universiti Malaya, Kuala Lumpur 50603, Malaysia; retnagowri@ummc.edu.my; 5 Advanced Medical and Dental Institute, Universiti Sains Malaysia, Penang 13200, Malaysia; soohuat@usm.my; 6 Department of Biomedical Sciences, Faculty of Medicine and Health Sciences, Universiti Putra Malaysia, Serdang 43400, Malaysia; fezah@upm.edu.my; 7 Department of Bioprocess and Polymer Engineering, School of Chemical and Energy Engineering, Faculty of Engineering, Universiti Teknologi Malaysia, Johor Bahru 81310, Malaysia; r-rosnani@utm.my; 8 Institute for Research in Molecular Medicine (INFORMM), Universiti Sains Malaysia, Penang 11800, Malaysia; boonyin@usm.my; 9 Department of Medical Microbiology & Parasitology, Faculty of Medicine & Health Science, Universiti Putra Malaysia, Serdang 43400, Malaysia; rishcapri@gmail.com; 10 Centre for Tissue Engineering and Regenerative Medicine, Faculty of Medicine, Universiti Kebangsaan Malaysia, Cheras, Kuala Lumpur 56000, Malaysia; 11 Department of Biomedical Sciences, Faculty of Medicine and Health Sciences, Universiti Malaysia Sabah, Kota Kinabalu 88400, Malaysia

**Keywords:** natural extract, cancer, cellular signaling pathways, target identification

## Abstract

*Andrographis paniculata* is a local medicinal plant that is widely cultivated in Malaysia. It is comprised of numerous bioactive compounds that can be isolated using water, ethanol or methanol. Among these compounds, andrographolide has been found to be the major compound and it exhibits varieties of pharmacological activities, including anti-cancer properties, particularly in the lipid-dependent cancer pathway. Lipids act as crucial membrane-building elements, fuel for energy-demanding activities, signaling molecules, and regulators of several cellular functions. Studies have shown that alterations in lipid composition assist cancer cells in changing microenvironments. Thus, compounds that target the lipid pathway might serve as potential anti-cancer therapeutic agents. The purpose of this review is to provide an overview of the medicinal chemistry and pharmacology of *A. paniculata* and its active compounds in terms of anti-cancer activity, primary mechanism of action, and cellular targets, particularly in the lipid-dependent cancer pathway.

## 1. Introduction

To date, many plants in Malaysia have been scientifically proven to contain natural ingredients that are effective against certain diseases. Different parts of the same plant may exhibit different medicinal properties. To maximize these properties, different extraction methods can be used, and this also depends on the type of favorable chemicals present in the respective plants. Usually, natural ingredients derived from plants offer a rich source of chemical compounds that have many therapeutic benefits in modern medicine. The value of these contents could be assessed as new target therapeutic and nutraceutical molecules instead of focusing on one specific active ingredient. Interestingly, since these compounds and chemicals are natural plant derivatives, there are no known documented side effects [1]. In Malaysia, more than 2000 plants have been categorized as medicinal plants due to their bioactive compounds and effectiveness against certain diseases [2]. In addition, medicinal plants have also long been used as a source of medicine, particularly in underdeveloped nations where medications are sometimes unavailable or prohibitively expensive, forcing people to rely on traditional treatments. Starting in the late 1980s and continuing in recent years, there has been a significant increase in research into herbal plants as alternative medicinal agents for various ailments. As such, it is predicted that the herbal industry in Malaysia will rise 15% per year, with a market value of USD 1.6 billion per annum [3], due to the rich biodiversity in its tropical rainforest. Among this biodiversity, *Andrographis paniculata* has received growing attention due to its exhibition of a wide spectrum of pharmacological properties.

### 1.1. Andrographis paniculata

*Andrographis paniculata* (*A. paniculata*) is an annual herb that belongs to the family of Acanthaceae. It is also commonly known as the “king of bitter” due to its extreme level of bitterness [4]. It usually grows up to one meter, with branches of dark green, slender stems. Their leaves are approximately 8 cm long and 2.5 cm wide, lance-shaped with hairless blades and a smooth surface. The flowers appear separately as tiny, pink, upright panicles, containing five green sepals along with white corolla. The fruit capsule is about 2 cm long and 3 mm wide, accommodating many yellow-brown seeds [5]. This plant is widely cultivated in Southeast Asia due to its ability to grow easily in a shady climate and most soil types [6]. Traditionally, it is used to treat general inflammation, diabetes, jaundice, cough, skin infections, and snakebites (Hossain et al., 2021) and, in Malaysia, it is widely known for its potential as an anti-inflammatory, anti-bacterial, anti-pyretic, and immunosuppressive agent [7]. Bioactive compounds such as polyphenols, andrographolide, and diterpenoids are found in *A. paniculata*. Furthermore, a few rare types of noriridoids, more than 55 types of ent-labdane diterpenoids, 8 quinic acids, 30 flavonoids, and 4 xanthones have also been isolated from these plants [5].

### 1.2. Phytochemistry

Different parts of *A. paniculata* are comprised of different compounds. The compound to be isolated also depends on the season of harvest, type of extraction method, and region of plant growth [8]. The ideal component based on the therapeutic use can be separated through bioactivity-guided chromatographic fractionation, such as silica gel chromatography or recrystallization [9]. For instance, it is impossible to isolate flavonoids via water extraction, but it is easy to isolate terpenoids, saponin, steroids, alkaloids, tannins, and flavonoids through ethanol extraction [8]. The majority of the active compounds of *A. paniculata* can be extracted from the leaf, which is 2.3% andrographolide. Similarly, leaves also contain other acidic components such as neo-andrographolide and diterpenoids; viz. deoxyandrographolide-19ß-Dglucoside [10].

Flavones, such as 5-hydroxy-7,8,2′,3′-tetramethoxy, andrographolide, andrographon, stigmasterol, andrographosterin 14-deoxy-11,12-didehydroandrographolide, andrographan, and homoandrographolide, as well as apigenin-7,4′-di-O-methyl ether, which are the main flavonoids, can be obtained from the roots of *A. paniculata* [11]. However, polyphenols, andrographolide diterpenoids, and 5,7,2′,3′-tetramethoxyflavanone, which are responsible for a wide range of biological activities, can be derived from any part of the plant [12]. Aside from these, other compounds, such as chalcone glucoside, flavone glycoside, androechin, acylated flavone glycosides, and oxygenated flavones, can also be derived from *A. paniculata* [13].

The presence of α-alkylidene γ-butyrolactone, the D12 (13) double bond, C-14 hydroxyl, and the D8 (17) double bond in andrographolide is said to be responsible for the cytotoxic activity towards several type of cancer cells [14]. Surprisingly, only methanol extraction of *A. paniculata* into a dichloromethane fraction was found to prevent cancer cell proliferation. Further fractionation of the methanolic extracts into andrographolide demonstrated the greatest anti-cancer efficacy [9]. However, the aqueous extract of *A. paniculata* outperformed the ethanol extract in terms of free radical scavenging, xanthine oxidase inhibition, and anti-lipid peroxidation [15].

## 2. An Outlook on the Lipid-Dependent Cancer Pathway

Cancer cells are notorious for their ability to alter metabolism in order to adapt to their need to sustain rapid proliferation. Lipid catabolism plays a major role in the continuous supply of the ATP and macromolecules needed for the proliferation of cancer cells [16], enhancing the lipid dependency of tumor cells in developing cancers. Lipid-dependent cancer can be defined as the development of cancer due to abnormal levels of lipids and their metabolites. Abnormal levels of lipid catabolism and metabolism can induce invasion of cancer cells via numerous pathways. For instance, studies have shown that high levels of fatty acid synthase in the cell membrane are commonly seen in cancer cells. Researchers speculate that lipid catabolism, metabolism, and transport have an influence on the emergence of cancer, but this is not specific to the type of cancer. Abnormalities in lipid composition can stimulate carcinogenesis in any organ [17].

### 2.1. The Association between Excessive Lipid Intake and Cancer Development

A high fat diet (HFD) has been shown to activate oncogenes by triggering inflammatory response such as excessive release of pro-inflammatory cytokines, including interleukin (IL)-1α, IL-1β, IL-6, and tumor necrosis factor (TNF)-α [18]. Elevated levels of macrophage markers, such as toll-like receptors 4 (TLR4) [19] CR4, EGF-like module-like mucin receptor-like hormone 1 (EMR1), immune complexes/TLR ligands [20], IL-10, adipose inflammatory cytokine [21], and decreased level of microRNA (miR)-130a, are evidence of the presence of cancer due to excessive HFD intake [22]. HFD-induced inflammation responses have the capacity to suppress the cancer cell apoptotic process by activating the signaling pathway, as mentioned previously, and increasing recruitment macrophages to epithelial tumors, thus promoting the angiogenesis and cell proliferation of cancer cells.

High consumption of dietary fat is linked to intestinal carcinogenesis via the activation of monocyte chemo-attractant protein (MCP)-1/CC chemokine receptor 2 (CCR2). Consumption of HFD increases the level of triglyceride production, and when this exceeds apolipoprotein production it leads to the formation of hepatic lipidosis, microbiota dysbiosis, gut inflammation and even reorganization of the gut–brain vagal communication [23]. Altered microbial composition can disrupt the intestinal barrier by increasing the permeability of the intestinal epithelium, which favors bacterial translocation. This alteration activates the MCP-1/CCR2 signaling axis to attract tumor-associated macrophages [24]. As such, there is increased production of inflammatory mediators and decreased levels of short-chain fatty acids. According to the literature, short-chain fatty acids play an important role in immunomodulatory functions, as well as in maintaining intestinal homeostasis by providing fuel and strengthening the gut barrier function [25]. Furthermore, short-chain fatty acids may also trigger signaling cascades that govern gut immunological activities by signaling via cell surface G-protein receptors (GPCRs) [25]. The dysregulation level of short-chain fatty acids can induce the downregulation of GPCRs, thus decreasing AMP-dependent protein kinase and cell proliferation and further damaging the epithelial barrier function [25]. Without proper treatment, chronic inflammation and even initiation of cancer might occur afterwards.

In addition, decreased levels of miR-130a strongly correlate with HFD-induced cancer. Recent studies have shown that miR-130a is mainly targeted on the TGF-β/BMP signaling pathway. It functions to suppress the activity of the TGF-β/BMP signaling pathway and control cell reprogramming [26]. MiR-130a inhibits Smad4 expression by binding directly to the 3′UTR of Smad4 at the miRNA consensus sites of miR-34a, miR-146a, and miR-199a, which are also the negative regulators of Smad4 in cancer progression [27]. Moreover, HFD intake increases the expression of the fatty acid receptors that are located on the cell surface, such as B-scavenger receptor (CD36). CD36 is a transmembrane glycoprotein with many posttranslational modification sites that binds to a series of ligands, such as apoptotic cells, thrombospondin-1 (TSP-1), and fatty acids. As a result, it acts as a fuel for tumor metastasis, lipid uptake, and fatty acid oxidation. CD36 prevents angiogenesis in tumor microvascular endothelial cells by binding to TSP-1 and inducing apoptosis or blocking the vascular EGFR 2 pathway. Furthermore, tumor immune tolerance and cancer growth are attributed to CD36-driven lipid metabolic reprogramming and functions in tumor-associated immune cells [28]. Given the complexity of the metabolic link between HFD intake and tumorigenesis, characterized by abnormal expression of various genes and dysregulation of cytokines and signaling pathways, further elucidation of the molecular mechanism linking lipid metabolism and cancer development is essential for the development of novel biomarkers and therapeutic markers in cancer therapy.

### 2.2. The Influence of Lipid Catabolism in Cancer Pathway

Lipid catabolism depends on the metabolism of fatty acid synthase (FASN). FASN is responsible for the regulation of endogenous fatty acids. For the normal regulation process, FASN functions to transform dietary carbohydrates via nicotinamide adenine dinucleotide phosphate (NADPH), acetyl-CoA, and malonylCoA to saturated fatty acid chains [29]. However, in cases of high levels of dietary fat intake, there will be high levels of low-density lipoprotein (LDL) cholesterol in plasma [30] and elevations in the regulators of the lipogenic gene transcription factor, such as the sterol regulatory element-binding protein (SREBP) [31]. Increased levels of LDL eventually reduce the outflow of cholesterol, which further enhances the production of endogenous cholesterol and fatty acids. Excessive levels of cholesterol suppress the low-density lipoprotein receptor (LDLR) and FASN as they are not able to function as normal [32]. Suppression of LDLR enhances the formation of 3-hydroxy-3-methyl-glutaryl-CoA reductase (HMGCR), a transcription enzyme responsible for direct cholesterol synthesis [33]. Additionally, the presence of miR-33 in the introns of SREBP further prevents the loss of cholesterol by hindering the activity of cholesterol transporter genes such as ABCA1 and ABCG1 [34]. As a result, cholesterol accumulates in the intracellular space, which may induce Akt signaling [35] and promote caveolin-1 (CAV-1) expression [36]. Stimulation of this transduction pathway increases the gene expression of Akt isoforms, causing the oncogene to be apoptosis-resistant and impaired in differentiation, which can accelerate the progression of the oncogene cell cycle [37]. This causes the oncogene to be activated, thereby initiating the development of a tumor. Meanwhile, CAV-1 affects cell metabolism by regulating glycolysis, mitochondrial bioenergetics, glutaminolysis, fatty acid metabolism, and autophagy, all of which are related to cancer growth and carcinogenesis. The promoter methylation of the CAV-1 gene results in CAV-1 silence, fostering cancer cell proliferation [38].

Aside from this, the Warburg effect is also involved in cancer development associated with lipid synthesis. The Warburg effect can be described as dysregulation of the oxidative metabolism as seen by increased aerobic glycolysis and decreased oxidative phosphorylation. Due to this effect, the same glucose metabolites that are used for ATP formation are diverted into the pentose pathways for lipid synthesis [39]. As such, this increased level of atypical glucose metabolites may cause further proliferation of cancer cells via the Akt pathway, as mentioned above. In contrast, increased expression of CAV-1 absorbs glucose metabolites and directs the export of cholesterol to caveolae [36]. This promotes N-glycosylation of SREBP cleavage-activating protein (SCAP) and subsequent activation of SREBP-1 [40]. N-glycosylation is a mediator for the epidermal growth factor receptor (EGFR) signaling pathway and it is via this pathway that the progression of the tumor continues. EGFR is a transmembrane signaling protein that transduces extracellular signals and is part of the tyrosine kinase family of membrane-bound receptors [41]. EGF and TGFα are the endogenous ligands for EGFR. As such, EGFR binds to its ligands [42]. EGFR dimerizes with another EGFR and autophosphorylates following ligand binding, which results in a sequence of intracellular signaling events. This includes activation of tyrosine kinase in the cytoplasmic domain, which triggers the mitogen-activated protein kinase (MAPK), AKT, protein kinase C (PKC), and signal transducer and activator of transcription (STAT). This cascade of signaling events promotes the survival, proliferation, invasion, and apoptosis resistance of the tumor cell [43].

Abnormal levels of lipids may result in mononuclear inflammation and mitochondrial abnormalities [44]. Mononuclear inflammation may stimulate the release and expression of plasminogen activator inhibitor-1 (PAI-1) [45]. PAI-1 has a growth stimulatory effect in cancer cells, causing cyclin D3/cdk4/6 upregulation and cell cycle progression from G1 to S, initiating the progression of cancer cell developments. Through its anti-fibrinolytic activity, modulatory activity on cell adhesion, and uPA/uPAR activity, PAI-1 has an indirect modulatory effect on cancer cell development. PAI-1 retains the action of thrombin, which interacts with protease activation receptors (PARs) in tumor cells and promotes proliferation through PAR activation, by inhibiting fibrinolysis [46]. PAI-1, which is derived from tumors, facilitates the migration of inflammatory cells by interacting with LRP-1, as well as the synthesis of IL-8 and leukotriene B4 in alveolar epithelial cells. It also increases the number of fibrosis-inducing cells and macrophages in the body. Through the activation of p38MAPK and NFB in macrophages, as well as the transcriptional upregulation of IL-6, PAI-1 influences the polarization of monocytes/macrophages toward an M2 pro-tumorigenic role [47].

### 2.3. Influence of Lipid Transport in Cancer Pathway

The core of lipid transport is the ATP-binding cassette transporter A1 (ABCA1)-mediated transmembrane subtransport pathway. Oncogenic mutations or cancer-specific ABCA1 loss-of-function mutations decrease ABCA1 gene expression, which results in the compromise of ABCA1, enhancing the buildup of mitochondrial cholesterol and inhibiting the release of death-promoting molecules like cytochrome C, causing cancer cells to survive [48]. iR-183 functions as an oncogene, increasing intracellular cholesterol by targeting the 3′UTR of ABCA1 mRNA. Through degradation of ABCA1, MiR-183 stimulates colon cancer cell proliferation while remaining resistant to apoptosis. Similarly, decreased ABCA1 expression triggered by hypermethylation elevates cholesterol levels, stimulating cancer cell development [49].

Intracellular cholesterol acts as a substrate for androgen complex synthesis, which promotes oncogene activation via the regulation of AKT signaling. Hypermethylation silences ABCA1, resulting in elevated intracellular cholesterol levels, which contributes to cancer progression. ABCA1-accelerated cholesterol efflux is significant for LXR inhibition in carcinoma cells. LXRs are members of the nuclear receptor family that regulate the expression of genes involved in cholesterol and fatty acid homeostasis. Previous studies have suggested that LXRs may be potential targets for cancer therapeutics. For instance, LXR-623 has the ability to penetrate the blood–brain barrier and may selectively kill glioblastoma cells in LXRβ in a cholesterol-dependent manner, thereby contributing to tumor regression [50].

Aside from this, scavenger receptor B type 1 (SR-B1) binds with HDL with high affinity to mediate selective cellular uptake and efflux of HDL-cholesteryl ester from the lipoprotein core. Therefore, overexpression of SR-B1 is also implicated in the cholesterol metabolism of cancer cells [51]. Additionally, in lipid transport mechanism membrane proteins, extracellular matrix tissue, cholesterol distribution, and cell signaling have all been linked to CAV-1 cell endocytosis.

## 3. The Role of *Andrographis paniculata* in Lipid-Dependent Cancer Pathways

Plant-derived natural biocompounds, particularly andrographolide from *A. Paniculata*, possess anti-cancer properties affecting the lipid-dependent cancer pathway. *A. paniculata* has been shown to have anti-cancer effects on cancer cells by inhibiting cell proliferation and differentiation, promoting cell apoptosis, blocking cell cycle progression, and improving body immune response against cancer cells via multiple lipid-dependent pathways. For instance, andrographolide inhibits the production of ROS through the modulation of the protein kinase C (PKC)-dependent pathway by blocking the action of phorbol-12-myristate-13-acetate (PMA). This is an important element in the production of reactive oxygen species (ROS) for the PKC-dependent pathway of cancer formation. At the same time, during this blocking event, andrographolide prevents neutrophil adhesion and transmigration by downregulating expression of macrophage-1 antigen (Mac-1) [52]. Hindered neutrophils prevent the adhesion of tumor cells and β2 integrins to neutrophils, thus preventing the aggregation of tumor cells [53]. Ingestion of 1 mg / body weight of *A. paniculata* can decrease the expression of IL-6, IL-8, IL-1β, COX-2, synoviocyte, and nitric oxide synthase (NOS) in an animal model. These may induce anti-neoplastic potential and reduce inflammation of the tumor microenvironment, thereby preventing cancer cell proliferation [54].

On the other hand, a study by Yuan et al. (2018) reported that andrographolide effectively suppresses angiogenesis in human colorectal cancer cells. Andrographolide is known to inhibit the actions of STAT3, JAK1, and JAK2 phosphorylation in human cancer cells [55]. Angiogenesis plays an important role in the growth of cancer cells and chemokines such as IL-8 and acts on endothelial cells to promote this process. The effect of andrographolide has been demonstrated by antagonizing TNF-α-induced IL-8 through a blockade of NADPH oxidase/ROS/NF-ƙB and Src/MAPK/AP-1 signaling pathways [56]. The blockage of NF-ƙB and MAPK pathways was also observed in ndrographolide-treated lipopolysaccharide (LPS)-stimulated RAW264.7 mouse monocyte macrophage cells. Andrographolide demonstrates obvious anti-inflammatory properties in suppressing the phosphorylation of ERK1/2, JNK, and p38, which are important pathways triggered in inflammatory responses [57]. Suppression of nuclear localization and translocation of AP-1, STAT-1, and STAT-3 in LPS-mediated macrophages are the primary proofs for the inhibitory effect of andrographolide in JAK/STAT pathways [52], as the JAK2 polymorphism is attributed to central fat accumulation and lipid-induced cancers [58]. The Janus kinase-signal transducer and activator of transcription (JAK/STAT) pathway is essential for maintaining tissue and organ homeostasis. The AK/STAT pathway is activated when cytokines or growth factors bind to the receptor, resulting in dimerization and activation of the relevant JAK(s). The activation, in turn, makes the phosphorylate receptor’s tyrosine increase and recruitment of STAT occur. STAT is phosphorylated by tyrosine and forms dimers. The dimers begin nuclease binding to specific DNA targets to activate transcription [59]. Interestingly, andrographolide is known to inhibit the actions of STAT3, JAK1, and JAK2 phosphorylation in human cancer cells [55,60]. Previously, Xu and colleagues (2013) have postulated that STAT3 encourages lipolysis and impedes adipogenesis [61]; hence, the dysregulation of JAK/STAT pathway may encourage the development of lipid-associated cancer [62].

Andrographolide may suppress the oncogene v-Src-induced transformation and downregulate v-Src protein expression via the attenuation of the ERK1/2 signaling pathway [63] or by stimulating secretion of IL-2 and interferon-c to promote the formation of cytotoxic T lymphocytes towards cancer cells [64]. Similarly, the growth of cancer cells is prevented through the degradation of hypoxia-inducible factor 1 (HIF-1)-mediated vascular endothelial growth factor A. HIF-1 expression is vital for tumor growth and angiogenesis. This mechanism is associated with suppression of HIF-1α protein and gene accumulation via inhibition of the PI3K/Akt-mTOR pathway in cancer cells. This is because inhibition of PI3K/Akt-mTOR-HIF-1 decreases lactate and ATP concentrations as well as the expression of GLUT1, HK-2, and PFK-1 in cancer cells. As a result, glycolysis is suppressed, thereby leading to a reduction in the cancer cells’ survival [65]. Oncogenic activity initiates multiple pathways to enhance the utilization of lipids and provide an energy source, which is required throughout the metabolic stress; the phosphatidylinositol with AKT signaling is the signaling system that is most often dysregulated in human malignancies [29]. In normal cells, growth factors trigger PI3K/AKT downstream pathways and the mammalian target of rapamycin (mTOR) network to activate transcription factors and hypoxia-inducible factor–1 (HIF-1) and sterol regulatory element–binding protein (SREBP) to elevate glycolytic flux and synthesis of fatty acid [66]. The PI3K/AKT/mTOR pathway is important for regulating the cell cycle and various functions of cell development [67]. The hyperactivated PI3K/AKT/mTOR pathway eventually results in deviated and uncontrolled cell proliferation with potentially metastatic effects [68,69,70]. In human papilloma virus (HPV)-infected cervical cancer cells, PI3K/Akt/mTOR signaling modulates the virus–host cell crosstalk. The crosstalk relies on the cellular metabolic and oxygenation status, which regulates HPV oncogene expression and the phenotypic response of HPV-positive malignance cells to the oncoprotein suppression associated with the PI3K/AKT/mTOR pathway [71].

Andrographolide causes apoptosis in prostate cancer cells by suppressing secretion of IL-6, resulting in inhibition of STAT3 phosphorylation [72,73]. The phosphorylation suppression of the tyrosine of gp130 by andrographolide effectively inhibits the JAK/STAT pathway, resulting in apoptosis in human cancer cells [73]. Another antineoplastic mechanism deployed by andrographolide is the inhibition of the PI3K/AKT/mTOR pathway [74]. In breast cancer cells, HIF-1α proteins decreased due to downregulation of the PI3K/Akt/mTOR pathway [52]. Li and colleagues (2020) discovered that andrographolide facilitates downregulation of the PI3K/Akt/mTOR pathway and inhibits glycolysis, a vital process that is needed for the survival and proliferation of cancer cells [75]. Andrographolide is also known to inhibit the PI3K/Akt/mTOR pathway and suppress angiogenic regulators such as vascular endothelial growth factor (VEGF) [76]. VEGF plays a major role in suppressing anti-cancer response in cells, which is facilitated by hypoxia-inducible factor (HIF)-1 [77]. Tanmoy and colleagues (2019) found that *A. paniculata* extract suppressed the expression of transcription factors Sp1 and coactivator protein but enhanced the expression of transcriptional regulator Sp3 in skin cancer cell, thus averting HIF1α binding [78]. The prevention resulted in the binding of HIF1α with a hypoxia-response element, thus inducing the anti-cancer element of *A. paniculata*.

Andrographolide reduces the expression of adhesion molecules such as ICAM-1 by blocking TNF-α-induced Akt phosphorylation in tumor cells [79], thereby suppressing tumor cell metastasis [80]. In addition, andrographolide inhibits expression of cyclin-dependent kinase by stimulating cell-cycle inhibitory protein p27. This increases the expression of p53 bax and caspase-3 and decreases expression of bcl-2 [81]. Apoptosis of tumor cells can be accelerated by an increased level of an apoptosis promoter such as bax, while the decreased level of the apoptosis inhibitor bcl-2 increases, confirming the reduction of cells resistant to apoptosis in response to anti-cancer stimuli [82]. The ingestion of 2 g/kg/day *A. paniculata* extract was reported to enhance the enzyme activity of CYP-2C6/11, -1A1/2, -3A1/2, and the level of mRNA in an animal model [83]. Furthermore, through activation of certain elements such as pro-apoptotic Bcl-2 and caspase 8, release of cytochrome C from mitochondria, and activation of p53 phosphorylation through the ROS-dependent JNK pathway, *A. paniculata* extraction is able to stimulate apoptosis in tumor cells [84].

In addition, the presence of Andrographolide has been proven to have cytotoxic effects against tumor cells. This mechanism functions through the activation of the NF-kB signal transduction pathway without inducing pyroptosis [85]. The inhibitors of caspase-8 and apoptosis the FLICE-like inhibitory protein (FLIP) and the X-linked inhibitor of apoptosis protein (XIAP) are both inhibited by NF-kB. Inhibition of NF-kB reduces caspase-8. The level of caspase-8 increases proportional to andrographolide concentration, while the level of caspase-1 remains constant. This indicates that the cancer cells are destroyed by andrographolide via a programmed apoptotic pathway rather than pyroptosis. This ensures that sudden lyses are prevented, since caspase-1 is known to activate pyroptosis, which is an inflammatory lytic pathway [86]. As a consequence, via the upregulation of tissue inhibitor of metallopeptidase-1 (TIMP1), the expression of matrix metallopeptidase-7 (MMP-7) is hindered, preventing the invasion of cancer cells [87]. Moreover, the MMP-9 promoter region contains an NF-kB binding site, indicating that the NF-kB transcription factor can play a role in MMP-9 activation in cancer progression [88]. Similarly, andrographolide represses NF-kB by restricting TNF-, IL-6, macrophage inflammatory protein-2 (MIP-2), NO, and PGE2 formation by LPS/IFN, causing a drop in the expression of COX-2 [89].

Li et al., (2017) explain the underlying mechanism for the prevention of cancer cell migration due to *A. paniculata* consumption. Within 24 h of *A. Paniculata* exposure, they saw a substantial decrease in mRNA expression of TM4SF3 and MMP9 in a concentration-dependent manner, inhibition of expression of HER2, CXCR4, MMP2, and MMP9, and depreciating tumor nodules. At the same time, the inhibitory effects of Cisplatin + 5-FU on the number of tumor nodules were enhanced [88]. The observed outcome further explains another pathway of *A. Paniculata* in preventing cancer pathogenesis. Another study showed that *A. Paniculata* inhibits SDF1α, the CXCR4 ligand that stimulates enterochromaffin cell migration in the colon, and HER2. The combined inhibition of HER2 and CXCR4 resulted in a further reduction in primary tumor development, demonstrating *A. Paniculata*’s enhanced anti-tumor and anti-metastatic properties.

In addition, *A. paniculata* shows direct anti-cancer activity against cancer cells. It does so by promoting cell cycle arrest in the G0/G1 phase, stimulating cell cycle-inhibitory protein p27 and reducing the expression of cyclin-dependent kinase 4. Increased lymphocyte proliferation and IL-2 production are both evidence of andrographolide’s immunostimulatory activity. Andrographolide also increases TNF production and CD marker expression, which indicates increased lymphocyte cytotoxic activity against cancer cells [90]. Aqueous extract of *A. paniculata* increases the activity of antioxidant protection enzymes, including catalase, superoxide dismutase, and glutathione-S-transferase, while lowering glutathione levels. By lowering the levels of thiobarbituric-acid-reactive substances and increasing glutathione concentrations, the extract significantly inhibits lipid peroxidation [91]. The accumulation of phorbol-12-myristate-13-acetate (PMA)-mediated ROS and N formyl methionyl leucyl phenylalanine (fMLP)-stimulated neutrophils could be prevented through the intake of *A. paniculata* in an animal model [92]. This demonstrates andrographolide’s ability to scavenge free radicals to minimize oxidative stress and the formation of thiobarbituric acid-reactive substances.

Moreover, diterpenoid lactones, isolated from *A. paniculata*, have proven to be effective against the stimulation of cell differentiation in cancer cells. This is possible through the inhibition of Janus tyrosine kinase–signal transducer and activator of transcription, phosphatidylinositol 3-kinase, and NF-B signaling pathways and the suppression of heat shock protein 90, cyclins, cyclin-dependent kinases, MMP, and growth factors, which may trigger tumor suppressor proteins p53 and p21 [93]. As a result, proliferation, migration, and survival of cancer cells will be stopped.

**Figure 1:**
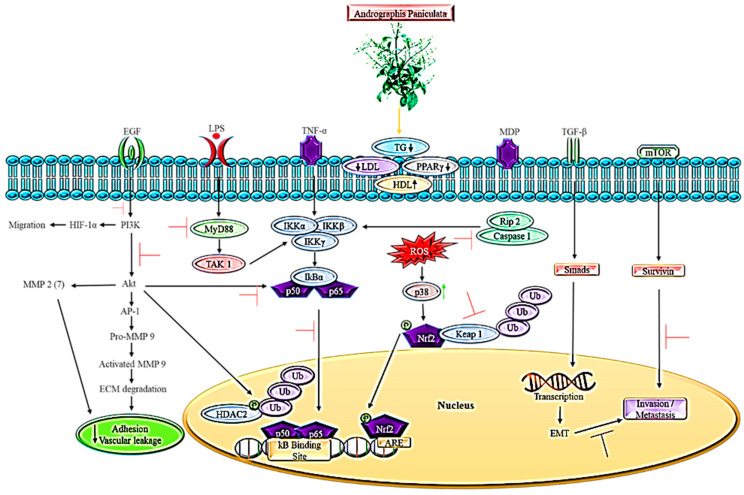
The possible mechanism used by *A. paniculata* in targeting lipid-mediated cancer pathways.

On the other hand, *A. paniculata* extraction binds covalently to the cysteine of p50 and Cys62 to prevent the NF-kB oligonucleotide from binding to nuclear proteins. As a result, this reduces the expression of E-selectin and subsequently inhibits E-selectin-mediated leukocyte adhesion by suppressing NF-kB activation in stimulated endothelial cells [57]. Cancer cells contain high levels of sialyl Lewis surface antigens, which interact with the adhesion molecules E- and P-selectin on activated endothelial cells, contributing to cancer cell adhesion to endothelial cells and tumor extravasation. By blocking E-selectin expression, andrographolide prevents cancer cells from adhering to activated endothelium [94]. Figure 1 shows the possible mechanisms used by *A. paniculata* in targeting the lipid-dependent cancer pathway.

## 4. Animal Model of Metastatic Spread in Conjunction with Pathways

In most cancer patients, metastization is the leading cause of death. There is an urgent need for models that more accurately mirror the biology and course of human cancer. In metastization, cancer cells are able to disperse from the initial tumor location to another organ through a complex process consisting of epithelial–mesenchymal transition, apoptosis elusion, invasion, cell migration, formation of new blood vessels and intravasation, and extravasation [95]. Understanding these metastatic mechanisms requires relevant models that depict the metastatic features of tumor cells. Animal-based in vivo metastasis models may better reflect the metastatic process and can be genetically modified to imitate human cancer.

The PI3K/AKT/mTOR signaling pathway is a well-known signaling pathway in cancer formation and metastatic spread [96]. PI3K/AKT/mTOR pathway activation has been linked to cell proliferation, progression, angiogenesis, therapy resistance, and invasion in various forms of cancer malignancy [97]. Recently, an in vivo study on mice by Tehranian and colleagues (2022) stipulated that the PI3K/AKT/mTOR signaling pathway is a critical phase in initiating brain metastatic development [98]. The experiment also found that the PI3K/Akt/mTOR pathway was elevated in brain metastasis compared to extracranial metastasis and primary tumors [98]. On the other hand, mutation in PIK3CA, a gene that is ubiquitously expressed in fat and implicated in breast cancer, alters the PI3K/AKT/mTOR pathway by activating the pathway [99]. PIK3CA mutations appear frequently and in multiple stages of cancer; in early primary tumor formation, late in the carcinogenesis process, right before or during invasion, and during metastatic lesions [100]. PI3KCA mutations in mice resulted in rapidly growing tumors in breast, cancer, and brain metastases [101]. In vivo, the PI3K/Akt/mTOR signaling pathway generated by caveolin-1 activation increased breast cancer movement, invadopodia development, and metastasis [102]. MicroRNA miR-20b and miR-451 are deregulated through the PI3K/AKT/mTOR pathway in gastric cancer [103,104]. Researchers Streleckiene and colleagues (2020) detected overexpression of miR-20b and a decline in miR-451a in mice with gastric cancer and stipulated that reverse expression of these microRNAs may contribute to a tumor-suppressive role [105].

The JAK/STAT pathway has been discovered to be critical in the dysregulation that promotes cancer cell growth and metastasis [106]. Dysregulation of the STAT3 gene promotes tumor growth as well as metastasis of cancer cells [107]. A high, constant, activated STAT3 encourages malignant tendencies in cells [107]. Over phosphorylation of STAT3 in the JAK/STAT3 pathway due to elevated neuropoietic cytokine activity in mice has been found to promote a metastatic effect in bone marrow cancer [108]. The overphosphorylation of STAT5 in the hyper-expressed JAK2/STAT5 pathway in mice has been found to result in a higher survival rate for prostate cancer, and hence has the possibility of being metastatic [109]. Similar findings were described by Cui and colleagues (2021) in mice with colorectal cancer [110]. Earlier, Chui and colleagues (2019) discovered that the activation of the JAK/STAT pathway occurs due to downregulation of TfR1 both in vivo and in vitro. Subsequently, the activation of the JAK/STAT pathway promotes cancer cell proliferation and potentially metastasis [111]. The researchers also discovered that tumors from TfR1 knockdown mice had slower growing rates and reduced weight compared to the control mice. Interestingly, in bone metastases, the activation of STAT3 is superior to the activation of STAT5, as demonstrated by Bottos et al. (2016). The authors also found that STAT3 activation enhances the spread of cancer to lungs and bones in mice [112]. Previously, Campbell and colleagues (2018) demonstrated that the elevation of isoform 133p53, which regulates the STAT1 gene, promotes tumor invasion by stimulating the JAK/STAT pathway. Elevated levels of 133p53 stimulate proinflammatory cytokines and complexes with its receptor to activate the JAK/STAT3 pathway, resulting in production of chemokines that encourage tumor cell migration [113]. Furthermore, the metastasis effect was drastically inhibited in a STAT6-silenced orthotopic 4T1 mice model with breast cancer. The mice showed lower metastatic tumor-associated macrophage (mTAM) colonization [114]. Lee and colleagues (2018) further demonstrated that IL-35 secreted by mTAM triggers the JAK2/STAT6/GATA3 pathway in orthotopic tumor model mice, which encouraged the spread of cancer cells by reversing the epithelial–mesenchymal transition [115].

## 5. Conclusions

In summary, this paper discussed the role of *A.paniculata* in lipid-dependent cancer pathways. Lipid metabolism is linked to each stage of cancer development. Cancer cells are assumed to tolerate the unnecessary metabolic effects of plasma lipid levels in order to preserve cancer development. Changes in the quantity and structure of lipid rafts as a result of cholesterol changes can have a direct impact on signal transduction, which causes oncogene activation. *A. paniculata* extracts, particularly andrographolide, target lipid metabolism in a variety of signaling pathways, resulting in beneficial therapeutic results based on tumor cell characteristics. The effect of andrographolide in regulating multiple lipid-dependent pathways has potential for future discoveries in anti-cancer therapy.

## Data Availability

Not applicable.
